# From Flight Miles to Community Immunity: How Loyalty Programs Can Revolutionize Vaccination in Saudi Arabia and the Gulf Cooperation Council (GCC)

**DOI:** 10.7759/cureus.97215

**Published:** 2025-11-19

**Authors:** Ali Alsaeed

**Affiliations:** 1 Infectious Disease Division, Department of Internal Medicine, Dammam Medical Complex, Dammam, SAU

**Keywords:** digital health, gcc healthcare, healthcare innovation, health loyalty programs, preventive care, public health, saudi arabia, vaccination strategy, vaccine science and policy, vision 2030

## Abstract

In a world where millions chase airline miles and loyalty points to secure that coveted flight upgrade or complimentary night at a luxury hotel, a fundamental question emerges: What if we could channel this same enthusiasm and motivational psychology toward one of the most critical aspects of our lives, our public health? Imagine a world where we don't wait for vaccination campaigns to reach us, but instead, we actively pursue them with genuine excitement, driven by an intelligent and innovative rewards system, much like we do with Emirates Skywards, one of the world's most successful loyalty programs.

Traditional vaccination campaigns, despite their considerable success, continue to face persistent challenges: vaccine hesitancy, difficulty reaching all population segments, and the need for intensive and ongoing awareness efforts. But what if we changed the equation? Instead of "pushing" people toward vaccination, we could "pull" them through an integrated ecosystem that transforms health maintenance into a rewarding and fulfilling journey.

## Editorial

A new vision: the "Healthwards" program inspired by Skywards

The idea is simple at its core yet profound in its impact: launching a national health loyalty program across Saudi Arabia and the Gulf Cooperation Council (GCC) countries, let's call it "Healthwards." This program would transform preventive healthcare, particularly vaccinations, into an experience akin to elite loyalty programs, built on a tiered and motivational structure.

Table 1 presents the proposed four-tier membership structure for the Healthwards program, outlining achievement criteria and corresponding benefits for each level.

**Table 1:** Healthwards membership tiers and benefits

Membership tier	How to achieve it	Proposed benefits and privileges
Blue Tier (new member)	Receive the first dose of any essential vaccine (such as childhood vaccinations or seasonal vaccines).	• Create a unified digital health record. • Access personalized health awareness content via the program app.
Silver Tier (fully vaccinated)	Complete the recommended vaccination schedule (such as two doses of COVID-19 vaccine or annual seasonal flu vaccine).	• All Blue-Tier benefits. • Priority booking for non-emergency appointments at health centers. • Discounts at partner pharmacies and fitness platforms.
Gold Tier (proactive health)	Maintain all recommended vaccinations up-to-date and complete an annual comprehensive medical check-up.	• All previous tier benefits. • 75% bonus health points redeemable for services. • Access to complimentary telemedicine consultations. • Free selection of certain service providers in specific cases.
Platinum Tier (health ambassador)	Achieve all Gold-Tier requirements, plus ensure family members (such as children) are vaccinated, and participate in blood donation campaigns.	• All previous benefits. • 100% bonus health points (double points). • Significant discount on health insurance premiums. • Complimentary fast-track access at major hospitals. • Complimentary Gold membership for one family member.

Why this model is perfect for Saudi Arabia and the GCC 

This idea arrives at an ideal moment, as the region embraces massive strategic transformations that place quality of life and innovation at the heart of national priorities.

Alignment With Vision 2030 and Beyond

The national visions across the region, particularly Saudi Arabia's Vision 2030, aim to build a vibrant society and thriving economy with a strong focus on digital transformation and preventive healthcare [1]. This program directly serves these objectives by enhancing public health using cutting-edge digital tools. The Kingdom has already invested over $50 billion in digital health initiatives, demonstrating unprecedented commitment to healthcare innovation [2].

A Digitally Native Society

Gulf residents enjoy high smartphone penetration rates and strong adoption of digital services. This makes implementing the program through a user-friendly digital platform both effective and widely acceptable. Research shows that private healthcare facilities in the Eastern Province of Saudi Arabia already demonstrate advanced digital health transformation capabilities, providing a strong foundation for such initiatives [3].

Culture of Rewards and Loyalty

Loyalty programs are not foreign to the region; they are an integral part of the retail, travel, and hospitality sectors. Applying this proven concept to healthcare will resonate positively with the public. Emirates Skywards itself was recently named the best loyalty program in the Middle East and Africa and ranked among the top 10 globally, proving the region's expertise in this domain [4].

Strategic Public-Private Partnerships

This model opens vast opportunities for collaboration between health ministries, private hospitals, pharmacies, insurance companies, and even other brands to offer joint rewards that benefit everyone. The GCC countries have already demonstrated proactive and effective implementation of comprehensive vaccination policies, making them ideal candidates for this innovative approach [5].

Scientific foundation and global methodology

This idea doesn't emerge from a vacuum; it's grounded in global research that has proven the effectiveness of incentives in promoting health behaviors. Recent studies have shown that incentives, whether financial or non-financial, can significantly increase vaccination rates [6]. Research published in prestigious scientific journals indicates that guaranteed non-cash rewards may be the most effective globally, as they avoid certain ethical issues associated with direct payment for healthcare services.

The evidence is compelling. Studies from Singapore found that incentives ranging from SGD 10 to 20 can effectively enhance vaccination rates while keeping costs manageable [6]. In the United States, a hypothetical financial incentive of $600 increased willingness to receive vaccines by more than 25%, while a $1,200 incentive raised it to over 30% [6]. However, the research also reveals an important nuance: in developed countries, educational programs and addressing mistrust in healthcare systems may be more effective than direct financial incentives alone [6]. Therefore, combining innovative incentives with continuous education and transparency represents the optimal strategy.

Successful healthcare loyalty programs worldwide have demonstrated remarkable results. CVS ExtraCare Plus combines immediate benefits with ongoing rewards, offering free delivery, a crucial advantage for patients with limited mobility [7]. UnitedHealthcare's Motion program encourages members to stay active by providing wearable fitness trackers and earning financial rewards for meeting daily walking goals, with rewards applied directly to health savings accounts [7]. These examples prove that when healthcare becomes rewarding, engagement skyrockets.

Implementation roadmap: from vision to reality

Transforming this concept into reality requires a strategic, phased approach that ensures sustainability and scalability across the GCC region.

Phase 1

Digital Infrastructure Development would involve creating a secure, HIPAA-compliant digital platform integrated with existing national health systems. This platform must support multilingual interfaces (Arabic and English at minimum) and provide a seamless user experience across mobile and web applications. The Kingdom's existing digital health transformation initiatives provide an excellent foundation for this development [3].

Phase 2

Strategic Partnership Formation requires engaging key stakeholders, including ministries of health, private healthcare providers, insurance companies, pharmaceutical chains, fitness centers, and retail brands. The partnership model should be mutually beneficial, where healthcare providers gain increased patient engagement, insurers benefit from healthier populations and reduced claims, and retail partners access a health-conscious consumer base.

Phase 3

Pilot Program Launch should begin in a specific region (such as Riyadh or Dubai) with a focused demographic group. This allows for testing, refinement, and demonstration of proof-of-concept before nationwide rollout. Key performance indicators should include enrollment rates, vaccination completion rates, patient satisfaction scores, and cost-effectiveness metrics.

Phase 4

National Expansion and Continuous Optimization involves scaling the program across all GCC countries while continuously analyzing data to improve reward structures, communication strategies, and partnership offerings. Machine learning algorithms can personalize recommendations and rewards based on individual health profiles and preferences.

Addressing challenges and ensuring ethical compliance

Any health loyalty program must navigate complex ethical and regulatory landscapes. The program must be designed from the ground up to comply with health information privacy regulations and anti-kickback statutes. Rewards should incentivize healthy behaviors without creating undue pressure or discrimination against those who cannot participate due to medical contraindications.

Transparency is paramount. Members must clearly understand how their health data is collected, used, and protected. The program should emphasize that vaccinations remain freely available to all citizens regardless of program participation, ensuring equity in healthcare access. The rewards serve to recognize and celebrate proactive health management, not to create barriers for those who choose not to participate.

The competitive advantage: why the GCC can lead

The Gulf region possesses unique advantages that position it to become a global pioneer in health loyalty innovation: strong governmental support for healthcare transformation, substantial financial resources for digital infrastructure investment, a young and tech-savvy population, existing expertise in world-class loyalty programs, and cultural values that emphasize family and community health all converge to create an ideal environment for this initiative.

Moreover, the region's experience in managing large-scale vaccination campaigns during the COVID-19 pandemic demonstrated impressive organizational capabilities and public cooperation [5]. Building on this momentum with a loyalty-based approach could establish the GCC as a model for preventive healthcare innovation that other nations will study and emulate.

Beyond vaccinations: a holistic health ecosystem

While vaccinations serve as the initial focus, the Healthwards program can evolve into a comprehensive wellness ecosystem. Future expansions could reward regular health screenings, chronic disease management adherence, healthy lifestyle choices (nutrition, exercise, sleep), mental health check-ins, and participation in community health initiatives. This holistic approach transforms the program from a vaccination tool into a lifelong health companion.

The economic benefits extend beyond individual health outcomes. A healthier population reduces healthcare system burden, increases workforce productivity, decreases insurance costs, and enhances overall quality of life-all contributing to the broader economic diversification goals outlined in national visions across the region [1].

Conclusion: the time for innovation is now

The transition from traditional healthcare models to proactive, incentive-driven approaches is no longer a luxury; it's a necessity for building healthier and more resilient societies. The Healthwards program can be the next step in the pioneering health transformation journey underway in Saudi Arabia and the Gulf countries, potentially becoming a global model for how to transform health obligations into rewarding and enjoyable lifestyle choices.

We stand at the intersection of technological capability, strategic vision, and societal readiness. The question is not whether loyalty programs can revolutionize public health; the global evidence confirms they can. The question is: Will the GCC seize this opportunity to lead?

Just as Emirates Skywards transformed how we think about travel loyalty, Healthwards can transform how we think about health commitment. The miles we earn today could become the immunity we build for tomorrow.
